# Vitamin D: A Potential Star for Treating Chronic Pancreatitis

**DOI:** 10.3389/fphar.2022.902639

**Published:** 2022-06-06

**Authors:** Meifang Zheng, Runping Gao

**Affiliations:** Department of Hepatic Biliary Pancreatic Medicine, The First Hospital of Jilin University, Changchun, China

**Keywords:** vitamin D, vitamin D receptor, chronic pancreatitis, fibrosis, inflammation

## Abstract

Chronic pancreatitis (CP) is a chronic inflammatory and fibrotic disease of the pancreas. The incidence of CP is increasing worldwide but the effective therapies are lacking. Hence, it is necessary to identify economical and effective agents for the treatment of CP patients. Vitamin D (VD) and its analogues have been confirmed as pleiotropic regulators of cell proliferation, apoptosis, differentiation and autophagy. Clinical studies show that VD deficiency is prevalent in CP patients. However, the correlation between VD level and the risk of CP remains controversial. VD and its analogues have been demonstrated to inhibit pancreatic fibrosis by suppressing the activation of pancreatic stellate cells and the production of extracellular matrix. Limited clinical trials have shown that the supplement of VD can improve VD deficiency in patients with CP, suggesting a potential therapeutic value of VD in CP. However, the mechanisms by which VD and its analogues inhibit pancreatic fibrosis have not been fully elucidated. We are reviewing the current literature concerning the risk factors for developing CP, prevalence of VD deficiency in CP, mechanisms of VD action in PSC-mediated fibrogenesis during the development of CP and potential therapeutic applications of VD and its analogues in the treatment of CP.

## 1 Introduction

Chronic pancreatitis (CP) is a multifactorial fibroinflammatory disease in which repeated episodes of pancreatic inflammation leads to extensive deposition of fibrotic tissue. The main clinical manifestations of CP are chronic pain, exocrine and endocrine pancreatic insufficiency, thereby declining life quality and shortening life expectancy. The pathophysiological processes of CP involve cellular injury, inflammation and fibrosis (Singh et al., 2019). The patients with CP in 5 years after diagnosis had a nearly eight-times increased risk for pancreatic cancer with a dismal prognosis (Kirkegård et al., 2017). The incidence and prevalence of CP are on the rise and extensive investigation on the treatment of CP has been done. However, there is still no effective treatment other than active care (Beyer et al., 2020). To explore agents that can be used for prevention or treatment of CP is needed urgently.

Vitamin D (VD) is a steroid hormone that has an important role in regulating body levels of calcium and phosphorus. It was initially widely used in skeletal system disorders because of its anti-rickets effect. Over the last several years, VD has been demonstrated to have pleiotropic effects including the regulation of cell proliferation, differentiation, apoptosis and autophagy as well as antagonizing inflammatory, fibrosis and cancer (Pike and Christakos, 2017; Golpour et al., 2019). Therefore, it has been also considered to be a promising therapeutic agent for non-skeletal system diseases such as cardiovascular disease, diabetes, cancer, infection, and autoimmune diseases (Jeon and Shin, 2018; Grant et al., 2020; Harrison et al., 2020; de la Guía-Galipienso et al., 2021). These exciting results inspire people to explore the correlation between VD and CP, and the potential therapeutic effects of VD in CP.

Previous epidemiological studies and clinical observations have found that VD deficiency is prevalent in patients with CP (Martínez-Moneo et al., 2016), but the correlation between VD level and the risk of CP remains controversial (Klapdor et al., 2012; Hoogenboom et al., 2016; Martínez-Moneo et al., 2016; Olesen et al., 2017). Several experimental studies have assessed the potential therapeutic benefits of VD in pancreatitis despite the therapeutic mechanism is not fully elucidated (Sherman et al., 2014; Bläuer et al., 2015; Kang et al., 2018; Wallbaum et al., 2018; Kang et al., 2021). VD analogue has been shown to suppress pancreatitis and the tumor stroma of pancreatic ductal adenocarcinoma *via* inhibiting pancreatic stellate cells (PSCs) activation (Sherman et al., 2014; Kang et al., 2021). Numerous studies are underway to elucidate the molecular mechanisms of VD/VD receptor (VDR) actions which involve in pancreatic and extra-pancreatic diseases. Some signaling pathways of VD/VDR in CP have been described, but their exact mechanisms need to be further clarified. Here we provide an up-to-date overview on these specific aspects, to better understand the potential therapeutic value of VD in CP. To our knowledge, this is the first review in this field.

## 2 Chronic Pancreatitis—Risk Factors and Pathogenesis

### 2.1 Risk Factors for Developing Chronic Pancreatitis

Excessive alcohol abuse is the most common cause of CP, affecting 42%–77% of patients with CP (Singh et al., 2019; Beyer et al., 2020). It has been also reported that the risk of developing CP in people with a long history of alcohol consumption was significantly higher than those not drinking (Singhvi and Yadav, 2018). Regular tobacco use is also a high risk factor of developing CP and there is a high prevalence (approximately 60%) of tobacco smoking among patients with CP (Beyer et al., 2020). Furthermore, the high risk of CP caused by smoking exhibits in a dose-dependent manner or in a combination with other risk factors, such as alcohol consumption (Rebours et al., 2012). Quitting smoking or alcohol or both can substantially reduce the risk of CP progression (Nikkola et al., 2013).

Additionally, several variants in genes including trypsin dependent and independent variants are also associated with CP, especially with idiopathic CP. These mutated genes include human cationic trypsinogen (*PRSS1*), pancreatic secretory trypsin inhibitor (*SPINK1*), chymotrypsin C (*CTRC*), cystic fibrosis transmembrane conductance regulator (*CFTR*), carboxypeptidase A1(*CPA1*) and claudin 2 (*CLDN2*) genes (Beyer et al., 2020). Other etiological risk factors include pancreatic duct obstruction, hypertriglyceridemia, hypercalcemia, IgG4-related disease, and chronic kidney disease (Singh et al., 2019; Beyer et al., 2020). CP is a multifactorial fibroinflammatory disease and its occurrence and progression can be usually promoted by multiple risk factors (HM et al., 2021).

### 2.2 Pathophysiology of Chronic Pancreatitis

The pathological features of CP are inflammatory cell infiltration, acinar atrophy, and pancreatic fibrosis. Pancreatic fibrosis is a pathological process characterized by the initial events of cellular damage and inflammatory cell infiltration, the involvement of multiple cytokines and inflammatory mediators, and the mediation of complex signal pathways, which in turn leads to PSC activation and extracellular matrix (ECM) production. Therefore, PSC plays a critical role in pancreatic fibrosis during the development of CP.

#### 2.2.1 Cellular Injury

In normal pancreas, acinar cells play an important role in the synthesis and secretion of digestive enzymes. Ethanol damages acinar cells through oxidative metabolite acetaldehyde and non-oxidative metabolite fatty acid ethyl ester, both of which can also damage pancreatic duct cells and PSCs. Damaged acinar cells can induce the activation of transcriptional activator nuclear factor—kappa B (NF-κB) and the expression of pro-inflammatory cytokines resulting in the activation of inflammatory cascade and necro-inflammatory response (Clemens et al., 2016). Smoking causes acinar cell damage due to the toxic metabolites of nicotine. Additionally, the premature or increased intrapancreatic activation of trypsinogen due to variants in the *PRSS1*, *SPINK1*, and *CTRC* genes is the initial step of CP, which damages acinar cells through several mechanisms, such as endoplasmic reticulum stress, oxidative stress and impaired autophagy. The trypsin independent variants in the *CFTR*, *CPA1*, and *CLDN2* genes also cause cell damage through different mechanisms (Witt et al., 2013; Giri et al., 2016).

#### 2.2.2 Inflammation

**FIGURE 1:**
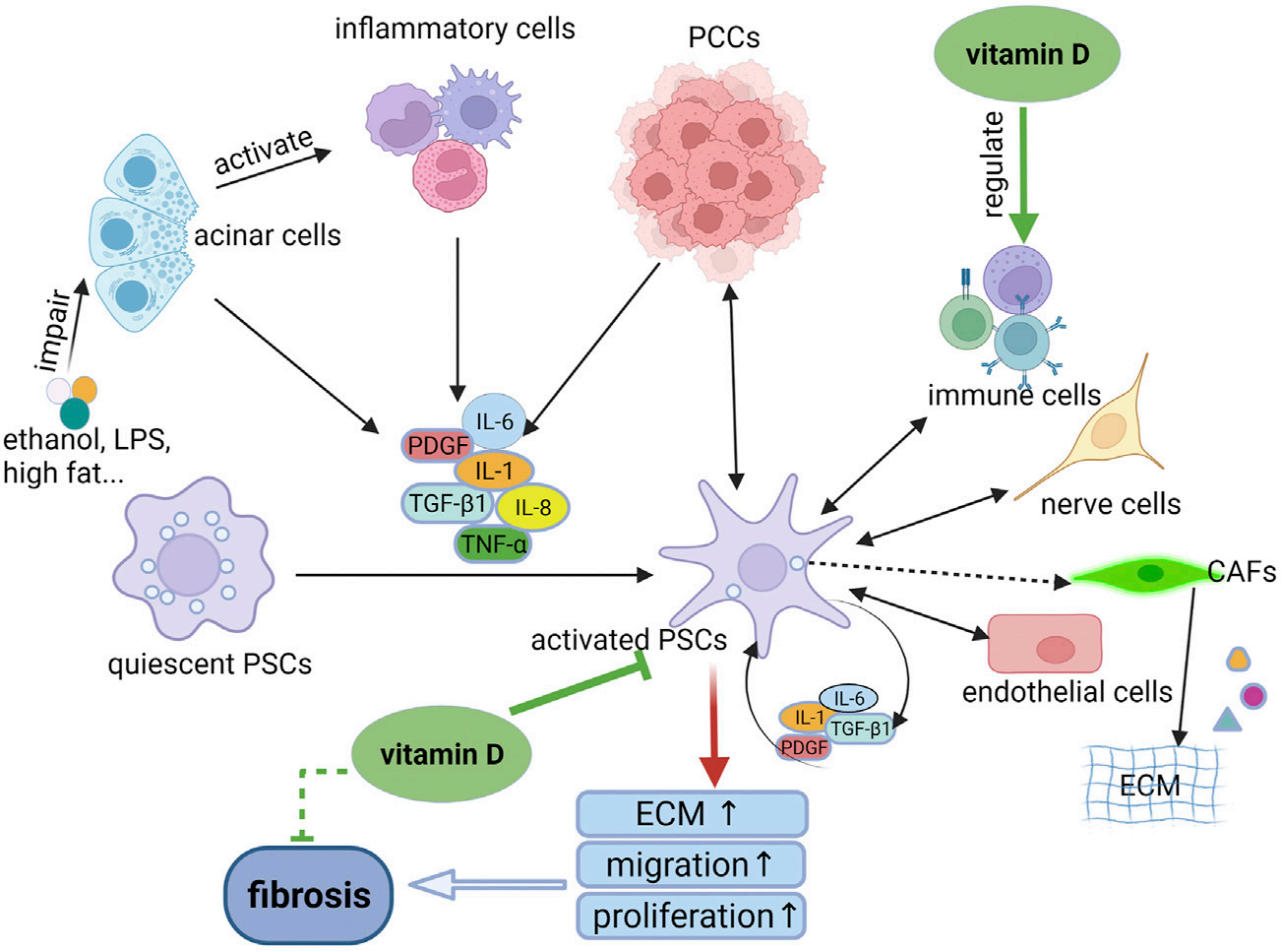
The mechanism of PSC activation and the role of vitamin D in the process. When the pancreas is injured by ethanol, LPS or other factors, the damaged acinar cells can activate inflammatory cells to release pro-inflammatory cytokines which in turn activates quiescent PSCs to become activated phenotypes through paracrine stimuli. The activated PSCs can secrete cytokines to activate PSCs continuously through autocrine stimuli, resulting in pancreatic fibrosis. In addition, the activated PSCs can interact with other cell types, such as PCCs and immune cells, mediating the persistent inflammatory environment. Whereas, vitamin D can inhibit the activation and proliferation of PSCs, thereby reducing the synthesis of ECM. In addition, vitamin D play an anti-inflammatory and anti-fibrosis role *via* regulation of immune cells. LPS, lipopolysaccharide; PSCs, pancreatic stellate cells; ECM, extracellular matrix; PCCs, pancreatic cancer cells; CAFs, cancer-associated fibroblasts.

Inflammation is mediated by cytokines, chemokines, and adhesion molecules. In the early stage of CP, injured acinar cells activate the key inflammatory cells such as macrophages, granulocytes and lymphocytes. All these cells then release a large number of proinflammatory cytokines, such as IL-1, IL-6, IL-8, tumor necrosis factor-alpha, transforming growth factor-beta 1 (TGF-β1), and platelet derived growth factor (PDGF). These proinflammatory cytokines can activate PSCs *via* paracrine stimuli. Meanwhile, the activated PSCs can also secrete cytokines for sustained activation of PSCs *via* autocrine stimuli. The sustained activation of PSCs leads to greater synthesis of ECM than degradation, eventually resulting in pancreatic fibrosis (Jin et al., 2020; Kandikattu et al., 2020; Zheng et al., 2021) (Figure 1). Additionally, NF-κB and activator protein 1 (AP-1) are important transcriptional factors that are involved in inflammatory responses. These two factors play an important role in initiating the inflammatory cascade in CP (Kandikattu et al., 2020).

Previous *in vivo* studies have demonstrated that T cells and macrophages are the predominant immune cell types in the pancreas of CP (Sun et al., 2018; Kandikattu et al., 2020; Zheng et al., 2021). Pancreases from mice CP models and patients were infiltrated by M2 macrophages instead of M1 macrophages. The M2 macrophages can effectively activate PSCs *via* a “feedforward” process, suggesting that macrophages play a key role in the fibrogenesis of pancreas (Xue et al., 2015). Increased lymphocytes have been observed in pancreatic tissue samples from patients with CP, thereinto, CD8^+^ T cells that reside between the pancreatic parenchyma and the fibrotic region are considered as key contributors to disease severity, CD8^+^ T cell- or NKT cell-mediated cytotoxicity may play an important role in the pathogenesis of CP (Bhatia et al., 2020). Moreover, mast cells, dendritic cells, eosinophils, monocytes, and B cells are also involved in inflammation of CP (Kandikattu et al., 2020).

#### 2.2.3 Fibrosis

PSCs are unique resident cells in the pancreas and play important roles in both the healthy and diseased pancreas. The activation of PSCs is a central link in pancreatic fibrogenesis (Bynigeri et al., 2017; Beyer et al., 2020; Li et al., 2022). PSCs can be activated by multiple triggers, such as ethanol and its metabolites, hyperglycemia, oxidative stress, cytokines, chemokines and stress, and then secrete excessive ECM, which causes interlobular and intralobular fibrosis. Advanced fibrosis can cause pancreatic exocrine and endocrine insufficiency. Among the cytokines, TGF-β1 is the most important driver of pancreatic fibrogenesis by promoting the activation of PSC and the production of ECM (Xu et al., 2017; Li et al., 2018b; Sun et al., 2018; Radoslavova et al., 2021; Zheng et al., 2021). Therefore, PSC is a potential target for antifibrotic therapy during the development of CP.

## 3 Vitamin D—Metabolism, Analogues, and Functions

### 3.1 Vitamin D Metabolism

**FIGURE 2:**
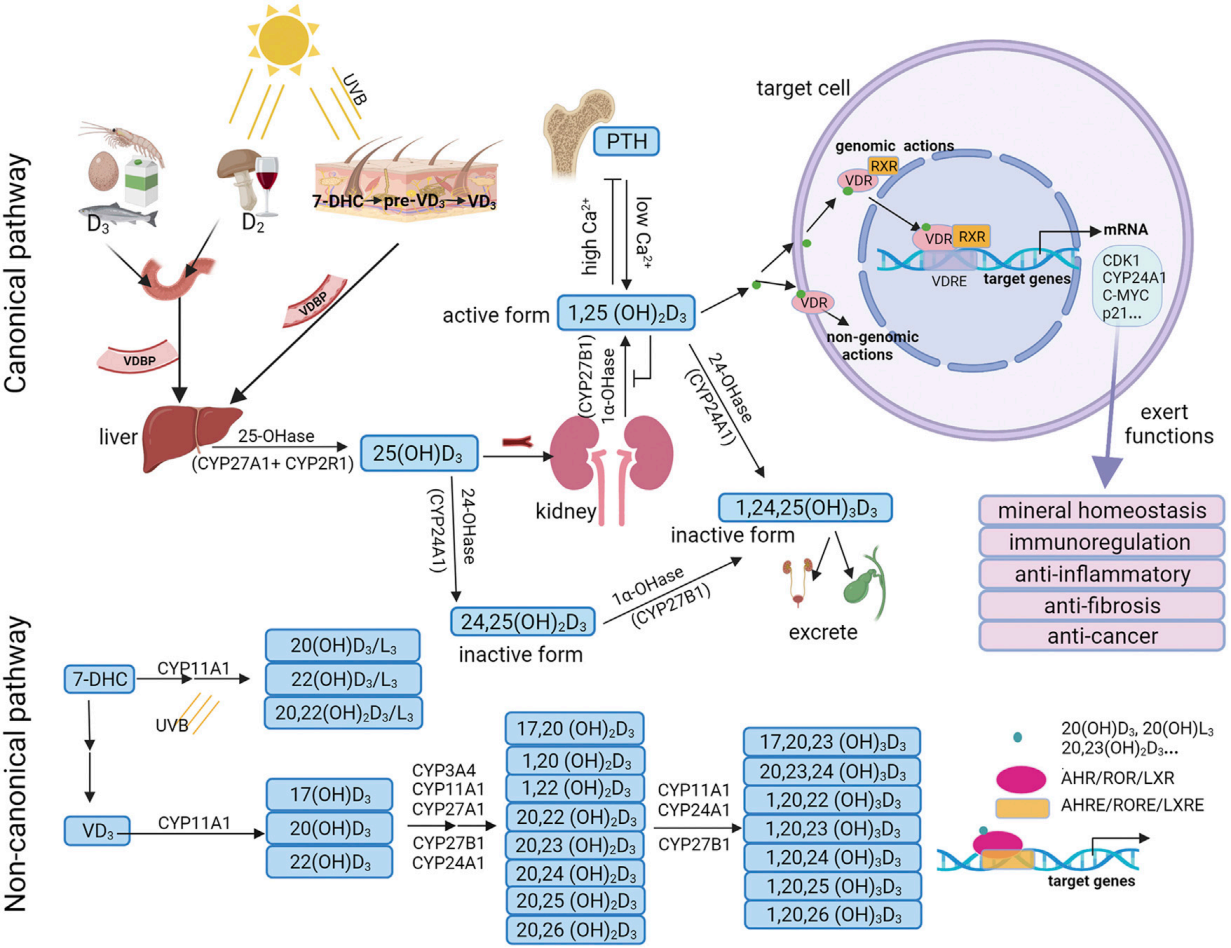
Vitamin D_3_ metabolism and biological functions. In the canonical pathway, vitamin D_3_ can be hydroxylated by CYP27A1/CYP2R1 and CYP27B1 to form 25(OH)D_3_, and is then further hydroxylated to the active form 1,25(OH)_2_D_3_. 1,25(OH)_2_D_3_ can bind with VDR/RXR and translocate to the cell nucleus, where it binds to VDRE to regulate the transcription of target genes. Besides, 1,25(OH)_2_D_3_ may bind with a membrane-associated receptor to mediate non-genomic actions. In the non-canonical pathway, 7-DHC and vitamin D_3_ are first hydroxylated by CYP11A1 and further hydroxylated by various cytochrome enzymes including CYP24A1, CYP27A1, CYP27B1, CYP2R1, CYP3A4, and CYP11A1 to form dihydroxy or trihydroxy metabolites. These bioactive metabolites selectively act on not only VDR, but also on alternative nuclear receptors such as AHR, RORs or LXRs and binds to AHREs, ROREs or LXREs to regulate the transcription of target genes. UVB, ultraviolet B; 7DHC, 7-dehydrocholesterol; VDBP, vitamin D-binding protein; PTH, parathyroid hormone; VDR, vitamin D receptor; RXR, retinoid X receptor; VDREs, VDR response elements; CDK1, cyclin dependent kinase 1; AHR, aryl hydrocarbon receptor; RORs, retinoic acid orphan receptors; LXRs, liver X receptors; AHREs, AHR response elements; ROREs, ROR response elements; LXREs, LXR response elements.

VD is a fat-soluble steroid hormone which was first known by its use in treating rickets in the 1920s. It can be obtained from the diet and by the action of sunlight on the skin. VD exists in two forms: VD_3_ and VD_2_. VD_3_ is endogenously produced in the skin and is the most utilized source of VD in animals. Exposure of the skin to ultraviolet B (wavelength 290–315 nm) rays results in the conversion of 7-dehydrocholesterol (7-DHC) to pre-VD_3_, which is followed by thermal isomerization to VD_3_. VD_2_ is produced by ultraviolet irradiation of ergosterol in fungi or yeast (Figure 2).

In the canonical pathway, VD (D_2_ or D_3_) is carried by VD-binding protein (VDBP) from the blood to the liver, where it is hydroxylated by a vitamin D-25-hydroxylase enzyme (25-OHase), such as CYP27A1 in the mitochondria or CYP2R1 in the microsome, to produce 25(OH)D3. 25(OH)D3 is the highest concentration of VD metabolite in the blood, with a half-life of approximately 15 days. Therefore, 25(OH)D_3_ is an effective indicator for the evaluation of the VD status in the human body (Hollis, 2005). In the kidney, 25(OH)D_3_ is further hydroxylated by 1α -hydroxylase enzyme (1α-OHase) (known as CYP27B1), to form 1α, 25-dihydroxyvitamin D_3_ (1,25(OH)_2_D_3_). 1,25(OH)_2_D_3_ is the most bioactive VD metabolite. 25(OH)D_3_ and 1,25(OH)_2_D_3_ can be catalyzed by CYP24A1 into inactive forms, 24,25(OH)_2_D_3_ and 1,24,25(OH)_3_D_3_, both of which are excreted through bile and urine (Wei et al., 2021) (Figure 2).

In the non-canonical pathway, VD_3_ can be activated by CYP11A1 to form primary hydroxylation products, such as 17(OH)D3, 20(OH)D_3_, and 22(OH)D_3_ (Slominski et al., 2015b). CYP11A1 is expressed not only in classical steroidogenic tissues such as the placenta, adrenal glands, and epidermal keratinocytes, but also in other organs and tissues such as the brain, gastrointestinal tract, thymus, and immune cells [reviewed in (Slominski et al., 2014a)]. Serum 20(OH)D3 and 22(OH)D_3_ levels were 30 and 15 times lower than 25(OH)D_3_ levels, respectively (Slominski et al., 2015b). These products can be further selectively hydroxylated by various cytochrome enzymes including CYP24A1, CYP27A1, CYP27B1, CYP2R1, CYP3A4, and CYP11A1 to form dihydroxy or trihydroxy metabolites (Slominski et al., 2012c; Slominski et al., 2015b; Slominski et al., 2015c; Jenkinson, 2019; Slominski et al., 2021b). The major CYP11A1-derived VD_3_ products are 20(OH)D_3_ and 20,23(OH)_2_D_3_ (Slominski et al., 2005; Tuckey et al., 2008; Slominski et al., 2012b; Slominski et al., 2016; Bocheva et al., 2021) (Figure 2). Additionally, VD_2_ and 7-DHC can also be hydroxylated by CYP11A1 to produce various metabolites, such as 20(OH)D_2_, 17,20,24(OH)_3_D_2_, 20(OH)D_3_/L_3_, 22(OH)D_3_/L_3_ and 20,22(OH)_2_D_3_/L_3_ (Jenkinson, 2019; Bocheva et al., 2021).

### 3.2 Analogues of Vitamin D_3_

1,25(OH)_2_D_3_ is the most bioactive form of VD and is also a potent agonist of the transcription factor VDR. VDR is a nuclear hormone that directly affects chromatin structure and gene regulation. The physiological function of VD is to control calcium homeostasis for maintaining bone mineralization. Moreover, VD can modulate innate and adaptive immunity, induce cell differentiation, apoptosis, and autophagy; inhibit cell proliferation, angiogenesis and metastasis; and regulate other cellular signaling processes (El-Sharkawy and Malki, 2020; Adelani et al., 2021; Murdaca et al., 2021; Pi et al., 2021; Poursoltani et al., 2021; Zhao et al., 2021; Bhutia, 2022; Zhou et al., 2022). Since VD levels obtained from diet are often insufficient and VD deficiency is associated with a variety of diseases, a daily supplement of at least 25 μg (1,000 IU) of VD is recommended to prevent VD deficiency (Holick et al., 2011). The variety and sales of VD supplementation are increasing in recent years.

Although VD is of great benefit to human health, overdosing with natural VD metabolites, such as 1,25(OH)_2_D_3_ and 25(OH)D_3_ may result in an increased hypercalcemia risk. Numerous VD analogues have been designed as potent VDR agonists with higher VDR binding affinity, but with lower hypercalcemia risk. So far, a few analogues have entered the market, such as, cholecalciferol, calcidiol [25(OH)D3], calcitriol [1,25(OH)_2_D_3_], and calcipotriol [22-ene-26,27-dehydro-1,25(OH)_2_D_3_], the latter of which is the most potential.

The majority of synthetic VDR agonists are derived from modifications of the 1,25(OH)_2_D_3_ at its side-chain, A-ring, C-ring, or triene system. There is also an increasing number of nonsteroidal mimics in recent years. These VD analogues have high binding affinity with VDR and maintain a good metabolic stability. Calcipotriol has been shown to have anti-inflammatory and anti-cancer effects in pancreatitis and pancreatic cancer *via* VDR pathway (Sherman et al., 2014). Currently, researches on VD analogues are conducted almost exclusively in academia, and many interesting methods for optimizing VDR ligands have not yet explored their limits.

In contrast to 1,25(OH)_2_D_3_ and 25(OH)D_3_, the CYP11A1-derived secosteroids, 20(OH)D_3_, and 20,23(OH)_2_D_3_ have no risk of causing hypercalcemia at pharmacological doses (Slominski et al., 2010; Wang et al., 2012a; Chen et al., 2014). In addition, 20(OH)D_3_ and 20,23(OH)_2_D_3_ have anti-fibrosis, anti-rheumatoid arthritis, and anti-cancer activities without hypercalcemia *in vivo* and *in vitro* (Slominski et al., 2012a; Slominski et al., 2013; Tang et al., 2013; Slominski et al., 2015c). This provides an alternative approach to investigate the therapeutic role of VD analogues.

### 3.3 Functions of Vitamin D_3_

The classical, hormonally-active dihydroxy form of VD_3_, 1,25(OH)_2_D_3_, plays multiple roles by regulating target genes through VDR pathway. VDR is an endocrine receptor and is a member of the superfamily of nuclear receptors (Carlberg, 2018). VDR is a novel protein that is able to bind 1,25(OH)_2_D_3_ and its analogues at sub-nanomolar concentrations in the human genome (Haussler et al., 1997). VDR is not only located in the skeletal system but also widely distributed in other tissues such as the small intestine (Battistini et al., 2020), kidney (Chokhandre et al., 2015), heart (Lin et al., 2019), lung (Wang and Jiang, 2021), pancreas (Wallbaum et al., 2018), liver (Triantos et al., 2021a), and immune cells (Wang et al., 2012b) as well as other cell types (Wang et al., 2012b). VDR is located in the cytosol of VD-target cells (Udomsinprasert and Jittikoon, 2019; Triantos et al., 2021b). Upon activation by 1,25(OH)_2_D_3_, the VD/VDR complex forms a heterodimer with the retinoid X receptor (RXR). The heterodimer then translocates into the cell nucleus and binds to specific DNA sequences known as VD response element (VDRE) which triggers the transcription of downstream genes (Christakos et al., 2016). VDRE is mostly located in the upstream of the transcription start site where VDR/RXR binds. The binding of VDR/RXR to VDRE promotes the recruitment of co-regulators that are necessary for chromatin remodeling and for the regulation of VDR/RXR-induced transcription of target genes (Christakos et al., 2016). Intriguingly, the VD degrading enzyme CYP24A1, as a target gene of VDR, can regulate VD homeostasis and thus can be used as a marker of VDR activation. 1,25(OH)_2_D_3_ has exhibited a wide range of biological functions mainly *via* VDR pathway, including the regulation of bone and calcium homeostasis, inflammatory response, immune response, cell proliferation, cell differentiation, and apoptosis (Christakos et al., 2016) (Figure 2).

In addition to the classical pathway of VD/VDR/RXR exerting biological effects, CYP11A1-derived products of VD_3_ such as 20(OH)D3, 1,20(OH)2D3, 20,23(OH)2D3, 20(OH)L3, and 20,22(OH)2L3 can also act on alternative nuclear receptors including aryl hydrocarbon receptor (AHR) (Slominski et al., 2018b), retinoic acid orphan receptors (RORs) (Slominski et al., 2014b; Slominski et al., 2017) or liver X receptors (LXRs) (Slominski et al., 2021a), thereby exerting pleiotropic effects including anti-fibrosis, anti-rheumatoid arthritis, anti-tumor, immunomodulatory, and photoprotection through regulation of target genes (Slominski et al., 2012a; Slominski et al., 2013; Tang et al., 2013; Slominski et al., 2014a; Slominski et al., 2015a; Slominski et al., 2015c; Tongkao-On et al., 2015; Slominski et al., 2017; Slominski et al., 2018a). AHR is the major receptor for 20,23(OH)_2_D_3_ and can also be activated by other CYP11A1-derived products of VD_3_ like 20(OH)D_3_ (Slominski et al., 2018b). Intriguingly, the expression of VDR and AHR are mutually exclusive in ovarian endometriosis. This may be explained by a divergence between a more pro-differentiation fate mediated by VDR versus a more pro-proliferation fate induced by AHR (De Pascali et al., 2021). 20(OH)D_3_ and 20,23(OH)_2_D_3_ can function as antagonists or inverse agonists of RORα and RORγ, providing new possibilities for skin and systemic regulation (Slominski et al., 2014b; Slominski et al., 2017). LXRs have been demonstrated to be the nuclear receptors for several VD_3_ and lumisterol (L_3_) derivatives, including 1,25(OH)_2_D_3_, 1,20(OH)_2_D_3_, 25(OH)D3, 20(OH)D3, 20(OH)L_3_, and 20,22(OH)_2_L_3_ (Slominski et al., 2021a) (Figure 2).

Except for genomic actions, some non-genomic actions of VD have been reported, which are mediated by cell surface receptors, but this still remains controversial (Hii and Ferrante, 2016; Bhattarai et al., 2017; Cui et al., 2017; Bollen and Atherton, 2021). Numerous studies have indicated that the non-genomic functions may not be important for VD-mediated transcription of target genes. The enzyme, protein disulphide isomerase family A member 3 (PDIA3) has been reported as a potential membrane-associated receptor for VD (Hu et al., 2019; Gisbert-Ferrándiz et al., 2020) and VD can stimulate the nuclear translocation of PDIA3-STAT3 (Hu et al., 2019). However, the significance of PDIA3 is still not elucidated because no binding site for 1,25(OH)_2_D_3_ has been confirmed. More researches are required to confirm whether there is VD-induced genomic actions or non-genomic actions *via* membrane receptors.

## 4 Vitamin D and Chronic Pancreatitis

### 4.1 Prevalence of Vitamin D Deficiency in Chronic Pancreatitis

The definition of VD deficiency of US Endocrine Society guidelines was the serum concentration of 25(OH)D_3_ less than 20 ng/ml (50 nmol/L), VD insufficiency was 21–29 ng/ml (50–74 nmol/L), and the satisfactory status of VD was 30–100 ng/ml (75–250 nmol/L) (Holick et al., 2011).

Patients with CP are often complicated with pancreatic exocrine insufficiency (PEI) of which steatorrhea, diarrhea, bloating, and weight loss are common symptoms. The main consequences of PEI are malnutrition and poor life quality. Deficiencies of fat-soluble vitamin, transferrin, and some kinds of micronutrient such as magnesium and zinc are common in PEI patients. Numerous studies have reported that the prevalence of VD deficiency in patients with CP ranging from 22% to 86.5% (Klapdor et al., 2012; Jøker-Jensen et al., 2020) (Table 1). Recently, our research found that 25(OH)D_3_ levels were significantly low in patients with alcoholic chronic pancreatitis as compared with healthy population.

**TABLE 1:** Overview of reports on vitamin D (VD) deficiency/insufficiency in CP.

Country	Participants	Testing indicators	Mean value	Criteria for deficiency/insufficiency	Prevalence of deficiency/insufficiency	References
Denmark	115 cases	VD (nmol/L)	57.8 ± 36.9 (10.0–175.0)	<25	22% (25/115)	Jøker-Jensen et al. (2020)
Germany	37 cases; 108 controls	25(OH)D_3_ (ng/ml)	CP: 15.6 ± 13.6 control: 17.5 ± 9.7	<30	CP: 94.2% control: 87% (*p* > 0.05)	Klapdor et al. (2012)
Germany	211 cases	25(OH)D_3_ (ng/ml)	20.2 ± 12	<20	56.39% (119/211)	Stigliano et al. (2018)
Ireland	62 cases; 66 matched controls	25(OH)D_3_ (nmol/L)	Not available	<50	CP: 58% control: 61.7% (*p* = 0.894)	Duggan et al. (2014)
United Kingdom	91 cases	VD	Not available	Not available	62.5% (55/88)	Min et al. (2018)
United States	100 pediatric cases	VD (ng/ml)	Not available	<20	5% (5/99)	McEachron et al. (2021)
India	72 TCP; 100 controls	25(OH)D_3_ (nmol/L)	CP: 24.0 (17.3–42.0) control: 27.5 (20.5–37.5) (*p* = 0.88)	<50	CP: 86% control: 85% (85/100) (*p* = 0.19)	Joshi et al. (2011)
Ireland	29 cases; 29 controls	25(OH)D_3_ (nmol/L)	CP: 31 control: 42 (*p* = 0.0126)	<50	CP: 69% (20/29) control: 62% (18/29) (*p* = 0.401)	Duggan et al. (2015)

VD, vitamin D; TCP, tropical calcific pancreatitis; CP, chronic pancreatitis.

A study from Denmark enrolled 115 consecutive CP outpatients and showed that micronutrient deficiencies in CP outpatients were varied and that VD deficiency (22%) was the most common micronutrient deficiency (Jøker-Jensen et al., 2020). A prospective multicenter study from Europe that enrolled 211 CP patients indicated 56% of VD deficiency (Stigliano et al., 2018). A study from United States, 62.5% (55/88) of patients had VD deficiency, and the rate of VD deficiency was higher in women as compared with men (67.3% vs. 54.5%, respectively) and was also higher in smokers versus nonsmokers (Min et al., 2018). Another study from United States showed that VD deficiency is also common in children. The total rate of VD deficiency and VD insufficiency is 27% in children with CP, and it is even higher (30%) after total pancreatectomy with islet auto-transplantation (McEachron et al., 2021). An earlier study from Germany reported that the prevalence of VD deficiency and insufficiency was 86.5% in patients with CP and 87% in normal controls, showing no difference between the two groups (Klapdor et al., 2012). A case-matched study from Ireland found no significant difference in serum 25(OH)D_3_ deficiency rates between CP patients and controls. Subgroup analysis demonstrated that VD levels were significantly lower in CP patients with osteoporosis than in CP patients without osteoporosis (Duggan et al., 2015). Taken together, VD deficiency is common in patients with CP, however, it is still unclear whether VD deficiency is a potential risk factor for the development of CP. Large-scale, high-quality prospective clinical studies are needed to elucidate the exact relationship between VD deficiency and the risk of CP.

### 4.2 Therapeutic Implications of Vitamin D

#### 4.2.1 *In Vivo* and *In Vitro* Studies

The activation of PSCs is a key step in the initiation and development of CP. Current *in vitro* studies mainly focus on the effect of VD on PSCs (Table 2). Primary PSCs from healthy mice were isolated and cultured. The activated cells were treated with VD_2_, VD_3_, and calcipotriol. The results showed that VD could increase lipid droplet storage, inhibit PSC activation, and decrease the expression of α-SMA and interleukin 6. However, VD didn’t have significant effect**s** on type 1 collagen (Col1) and TGF-β1 production (Wallbaum et al., 2018).

**TABLE 2:** Summary on the role of vitamin D in CP from *in vivo* and *in vitro* studies.

Function	Biological effects	References
Inhibition of activation of PSCs	↑Lipid droplet ↑VDR expression	Sherman et al. (2014), Wallbaum et al. (2018)
Anti-inflammatory	↓Pro-inflammatory cytokines	Wallbaum et al. (2018)
Anti-fibrosis	↓ECM	Sherman et al. (2014), Bläuer et al. (2015), Kang et al. (2018), Wallbaum et al. (2018), Kang et al. (2021)
Anti-proliferation	↓PSCs activation ↓PSC number ↑cyclin-dependent kinase inhibitors p21/p27 ↑cell cycle arrest at the G (1)/S checkpoint	Sherman et al. (2014), Bläuer et al. (2015), Kang et al. (2018), Wallbaum et al. (2018), Kang et al. (2021)
Induction of differentiation	↑VDR binding ↓SMAD3 binding ↓p-STAT3	Sherman et al. (2014)

VDR, vitamin D receptor; ECM, extracellular matrix; PSCs, pancreatic stellate cells.

In 2015, Finnish researchers investigated the anti-proliferation and anti-fibrosis effects of 1,25(OH)_2_D_3_ in PSCs. The activated PSCs were exposed to different physiological concentrations of 1,25(OH)_2_D_3_. The results showed that 1,25(OH)_2_D_3_ could inhibit the expression of fibronectin and Col1 and the proliferation of PSCs, with a positive correlation between anti-proliferation ability and 1,25(OH)_2_D_3_ concentrations. 1,25(OH)_2_D_3_ could also promote the expression of VDR in PSCs (Bläuer et al., 2015). A compound named as 9c has been recognized as one of the novel series of non-secosteriodal VD analogues to inhibit the expression of fibrotic genes and ECM deposition *in vitro* and *in vivo* (Kang et al., 2018) (Table 2).

#### 4.2.2 Clinical Studies

In addition to the above *in vitro* and *in vivo* studies, several observational studies and randomized controlled trials (RCTs) have also been conducted to investigate the therapeutic potential of VD in patients with CP. Due to the differences in population and the methods of biochemical analysis among these studies, the results of VD deficiencies in patients with CP versus controls are highly different (Table 3). Therefore, the existing studies are not enough to say whether VD deficiency is related to the risk of CP (Olesen et al., 2017).

**TABLE 3:** Summary on the roles of vitamin D (VD) in CP from clinical studies.

Country	Research type	Number of patients	Aim of the study	RR/HR/OR (95%CI, *p*)	Conclusion	References
Spain	Meta	548	To determine the prevalence of fat-soluble vitamin deficiency in CP patients	1.17 (0.77–1.78, *p* = 0.46) I^2^ = 0%	Fat-soluble vitamins deficiency is frequent in CP patients, but no significant increased risk of VD deficiency	Martínez-Moneo et al. (2016)
Netherlands	Meta	465	To determine the prevalence of VD insufficiency and deficiency in CP patients	1.14 (0.70–1.85, *p* > 0.05) I^2^ = 0%	High prevalence of VD insufficiency and deficiency in CP patients, but no significant difference between patients and healthy controls	Hoogenboom et al. (2016)
Germany	Meta	220	To analyze the results from RCTs of dietary interventions for CP patients and make further dietary recommendations	Not available	VD can improve VD deficiency in CP, while other nutritional support therapies have no evidence of effectiveness	Wiese et al. (2021)
Denmark	RCT	30	To assess intestinal absorption of cholecalciferol in patients with CP and fat malabsorption	*p* < 0.001	Daily VD supplementation increased 25(OH)D_3_ in CP patients compared to placebo, but this was not the case with weekly tanning bed sessions	Bang et al. (2011)
Denmark	RCT	30	To investigate the effect of changes in 25(OH)D_3_ and 1,25(OH)_2_D_3_ on Tregs in patients with CP with fat malabsorption	*p* < 0.05	Changes in VD significantly correlate with maturation of CD4^+^ and CD8^+^ Tregs	Bang et al. (2012)
India	RCT	40	To assess the relative efficacy of two different doses of VD in patients with CP with VD deficiency	*p* < 0.001	The 600,000 IU dose was more effective in achieving VD sufficiency over 6 months compared to 300,000 IU, but no longer after 9 months	Reddy et al. (2013)

CP, chronic pancreatitis; RCT, randomized controlled trial; VD, vitamin D; Tregs, regulatory T cells; RR, relative risk; HR, hazard ratio; OR, odds ratio; CI, confidence interval.

A latest systematic review and meta-analysis about nutritional management of CP enrolled five RCTs suggest that the supplementation of VD is a potential therapy for CP (Bang et al., 2011; Bang et al., 2012; Reddy et al., 2013) and that oral or intravenous VD can improve VD deficiency in patients with CP (Bang et al., 2011; Wiese et al., 2021). However, another RCT showed that 600,000 IU was more effective in achieving VD sufficiency over six months compared to 300,000 IU, but no longer after nine months (Reddy et al., 2013). Several other related RCTs are underway or completed but the results have not yet been published (Table 4).

**TABLE 4:** Clinical trials (http://clinicaltrials.gov/).

Clinical Trials.gov number	Conditions/diseases	Drugs	Intervention/treatment	Enrollment	Phase
*Unregistered Bang et al. (2011), Bang et al. (2012)	CP and fat malabsorption	Cholecalciferol	Cholecalciferol 1520 IU daily and calcium 800 mg weekly for 10 weeks, PO	30	Not applicable
*NCT00956839 Reddy et al. (2013)	TCP	Cholecalciferol	3,00,000/6,00,000 Units single dose, IM	40	VI
NCT02965898	CP	VD	100/10 μg daily for at least 7 years, PO	260	Not applicable
NCT01141998	CP with malabsorption syndromes	Calcium	400 mg two times daily week 0–10 and week 14–52, PO	27	Not applicable
NCT02108509	CP with osteopenia/osteoporosis	Not applicable	Not applicable	55	Not applicable

CP, chronic pancreatitis; TCP, tropical calcific pancreatitis; PO, oral intake; IM, intramuscular injection; VD, vitamin D.

## 5 Mechanisms of Vitamin D Action in Chronic Pancreatitis

### 5.1 Anti-Inflammatory and Anti-Fibrotic Effects

PSCs have similar physiological properties and functions to those of hepatic stellate cells (HSCs). So far, many *in vitro* studies by culturing PSCs or HSCs have confirmed the therapeutic potential of VD in pancreatic or liver diseases. Since HSCs were discovered earlier than PSCs, there are more studies on the mechanism of VD in anti-hepatic fibrosis as compared with anti-pancreatic fibrosis, thereby providing ideas and research methods for reference in the study of VD in anti-pancreatic fibrosis. For instance, an early study has shown that VD analogue calcipotriol antagonizes TGF-β -mediated pre-fibrotic gene expression in human HSCs through VDR/SMAD interaction (Ding et al., 2013). Based on some relevant researches on liver diseases, five signaling pathways of VD in anti-inflammatory and anti-fibrosis have been summarized as follows: 1) VD inhibit cyclin D1 expression, which is a key marker of the cell cycle, resulting in anti-proliferation of HSCs; 2) VD reduces SMAD3 occupancy at co-regulating genes, revealing an intersecting VDR/SMAD genomic circut that regulate hepatic fibrogenesis, thereby inhibiting TGF-β/SMAD-mediated pro-fibrotic effects; 3) VD inhibits the transcription of pro-fibrotic genes and activity of HSC by interacting with HSC-specific p62 and VDR; 4) VD activates VDR to bind with IKKβ by which the NF-κB transcriptional activity is impaired, thus reducing inflammatory response; 5) VD/VDR signaling attenuates TLR4-mediated inflammatory response by enhancing negative feedback regulation (Triantos et al., 2021b).

Many studies in rheumatoid arthritis, chronic obstructive pulmonary disease and cardiovascular disease have demonstrated that VD regulates the inflammatory microenvironment of the diseases through enhancement of p38 MAPK pathway, inhibition of NF-κB signaling and regulation of prostaglandin pathway (Moreno et al., 2005; Yang et al., 2015; Ishizawa et al., 2017; Gil et al., 2018; Wen et al., 2018; Derakhshanian et al., 2019; Qian et al., 2019; Yao et al., 2019; Zhou et al., 2019; Cimmino et al., 2020). 1,25(OH)_2_D_3_ can restrain macrophage-mediated inflammation processes by suppressing the AKT/NF-κB/COX-2 pathway in a carrageen-induced paw edema mouse model and it can also reduce the proliferation of fibroblast-like synoviocytes and the production of pro-inflammatory cytokines (IL-1β, IL-6, IL-8, and PGE2) in a rheumatoid arthritis rat model. (Wang et al., 2014; Fan et al., 2017).

### 5.2 Immunomodulatory Effect

The inflammatory cell storm plays an important role in the progress of CP in which many cell types including monocytes, macrophages, mast cells, and T cells are implicated (Kandikattu et al., 2020). Activated macrophages have been demonstrated as a critical regulator of inflammation and fibrosis that promote the production of collagen and fibronectin in PSCs *via* paracrine-cytokine signaling (Schmid-Kotsas et al., 1999). During the development of CP, local imbalances of T-cell subsets in inflammatory have also been observed (Schmitz-Winnenthal et al., 2010). The numbers of central memory T-cell subsets (CCR7^+^/CD45RA) were increased in blood samples from CP patients. Moreover, the increased CCR7^+^ memory T cells were not changed between unresected CP patients and subjects who had undergone pancreatic resection due to CP, suggesting that a persistent increase of central memory T lymphocytes may be important for maintaining the inflammatory process in CP (Grundsten et al., 2005). Therefore, targeting T cells may be a potential therapy to reverse the process of CP.

Various immune cells including macrophages, dendritic cells and lymphocytes express VDR constitutively or inductively, thus increasing immune response to antigens (von Essen et al., 2010; Scolletta et al., 2013). VD/VDR complex has been confirmed to play a role in T cell antigen receptor signaling and T cell activation as well as in the regulation of immune responses (von Essen et al., 2010; Di Rosa et al., 2011; Bang et al., 2012; Sarkar et al., 2016; Cantorna et al., 2019). Moreover, VDR agonists have significant inhibitory effects on macrophage- and monocyte-mediated inflammatory processes through controlling the expression and activities of VDR and CYP27B1 (Morán-Auth et al., 2013; Dionne et al., 2017; Martens et al., 2020; Wherry et al., 2021). 1,25(OH)_2_D_3_ or its analogue treated dendritic cells can modulate human autoreactive T cells *via* the selective induction of apoptosis (van Halteren et al., 2004; Gil et al., 2018; Vanherwegen et al., 2019). VDR agonists exert a significant suppression of inflammatory processes by switching the immune response from T helper 1 (Th1) to T helper 2 (Th2) dominance and by counteracting the self-enhancing inflammatory loop between immune cells and resident cells (Scolletta et al., 2013). VD suppresses the expression of IL-17 and IL-2 in CD4^+^ T cells and reduces CD8^+^ T cell-mediated cytotoxicity, which leads to an overall effect of blocking Th1-mediated responses (Meehan et al., 1992). Moreover, VD stimulates the development and differentiation of regulatory T cells (Tregs) and enhances their suppressive function (Treiber et al., 2015; Bogdanou et al., 2017; Di Liberto et al., 2019; Fisher et al., 2019). Likewise, B cell proliferation, plasmacyte differentiation, and immunoglobulin secretion are also influenced by VDR ligands perhaps *via* their effects on antigen-presenting cells or T cells (Chen et al., 2007; Vanherwegen et al., 2017b; Vanherwegen et al., 2017a).

### 5.3 Regulation of Proliferation

VDR agonists can inhibit the cell cycle of a variety cells, especially cancer cells. 1,25(OH)_2_D_3_ upregulates the expression of cyclin-dependent kinase inhibitors p21^(Waf1/Cip1)^ and p27^(Kip1)^, which plays a key role in G0/G1 phase cell cycle arrest and anti-proliferation (Wu et al., 2007; Irazoqui et al., 2014; Spath et al., 2017; Trump, 2018; Li et al., 2019; Gesmundo et al., 2020). A cross-talk between 1,25(OH)_2_D_3_/VDR non-genomic and genomic signaling at the level of MAPK activation has been demonstrated to reduce the proliferation of human osteosarcoma cells (Wu et al., 2007). The human p21^(waf1/cip1)^ gene has been recognized as a primary 1,25(OH)_2_D_3_-responding gene with at least three VDR binding promoter regions, in two of which are also co-localized with p53, therefore it is a primary anti-proliferative target for the VDR in the presence of 1,25(OH)_2_D_3_ (Saramäki et al., 2006; Li et al., 2017a). VDR is involved in the induction of p27^(Kip1)^ by VD_3_ and may interact with Sp1 to modulate the expression of target genes in LNCaP cancer cells (Huang et al., 2004). In addition, 1,25(OH)_2_D_3_ induces the expression of other cyclin-dependent kinase inhibitors, such as p15^(Ink4b)^ and p16 ^(Ink4a)^ (Chiang and Chen, 2013; Chen et al., 2019).

### 5.4 Induction of Differentiation

WNT/β-catenin signaling is activated in colon cancer cells which is associated with tumor cell malignancy and dedifferentiation (González-Sancho et al., 2020). 1,25(OH)_2_D_3_ can induce the transcription of genes involved in differentiation of bone, skin and brain cells by repressing WNT/β-catenin signaling (González-Sancho et al., 2020). VDR agonist can also reduce the amount of β-catenin binding to transcription factor T cell factor (TCF) by inducing the interaction between β-catenin and VDR (Larriba et al., 2013). E-cadherin is a transmembrane glycoprotein that connects epithelial cells together at adherens junctions. In normal cells, E-cadherin exerts its tumor suppressing role mainly by sequestering β-catenin from its binding to lymphoid enhancer factor (LEF)/TCF. 1,25(OH)_2_D_3_ induces high expression of E-cadherin and WNT inhibitor (DKK-1) leading to β-catenin nuclear export and relocation to the *adherens* junctions at the plasma membrane, thereby suppressing colonic carcinogenesis (Pendás-Franco et al., 2008; Larriba et al., 2013; Xin et al., 2017).

### 5.5 Induction of Apoptosis

VD has been confirmed to promote apoptosis in various cell types through different signaling pathways. 1,25(OH)_2_D_3_ induces apoptosis in adipocytes *via* activation of Ca^2+^-dependent calpain and Ca^2+^/calpain-dependent caspase-12 (Sergeev, 2009; 2020), providing a potential therapy for obesity. VD analogue paricalcitol reduced fibroid tumor size of nude mice through upregulation of apoptosis (Halder et al., 2014). 1,25(OH)_2_D_3_ induces apoptosis through inhibiting anti-apoptotic proteins BCL-2 and BCL-XL and inducing pro-apoptotic proteins such as BAX, BAK, and BAD in cancer cells (Díaz et al., 2000; Halder et al., 2012; Giammanco et al., 2015; Aslam et al., 2021), while VD shows an anti-apoptotic effect in peripheral blood mononuclear cells in systemic lupus erythematosus *via* increasing the expression of BCL-2 and decreasing the expression of BAX (Tabasi et al., 2015). VD also induces apoptosis of ovarian cancer cells through downregulating the activity of telomerase and the level of telomerase reverse transcriptase (Jiang et al., 2004). Furthermore, 1,25(OH)_2_D_3_ can also enhance the pro-apoptotic effects of gemcitabine, paclitaxel and cisplatin in squamous cell carcinoma through different pathways (Hershberger et al., 2001; Hershberger et al., 2002). 1,25(OH)_2_D_3_ enhances cisplatin-mediated cell apoptosis by decreasing the expression of ERK and AKT and increasing the expression of BAX, p21, and p27 in gastric cancer cells (Bao et al., 2014).

### 5.6 Induction of Autophagy

Autophagy is a cellular process in degrading of long-lived proteins and organelles and misfolded proteins in the cytosol for maintaining cellular homeostasis, which has been linked to many states of human health and disease (Xu et al., 2018; Zhang et al., 2021). Recently, VD has been demonstrated to alleviate ethanol-induced hepatotoxicity by enhancing autophagy (Yuan et al., 2021). 1,25(OH)_2_D_3_ has also been confirmed to improve hepatic steatosis by upregulating autophagy induced by ATG16L1 (Li et al., 2017b). Moreover, 1,25(OH)_2_D_3_ can increase cell viability and insulin secretion of rat insulinoma cells and protects cells from oxidative damage induced by streptozotocin *via* autophagy activation (He et al., 2019). *PRSS1*-related hereditary pancreatitis is characterized by episodes of acute pancreatitis and recurrent acute pancreatitis with frequent progression to CP, which damages acinar cells through several mechanisms including oxidative stress and impaired autophagy (Witt et al., 2013; Giri et al., 2016).

There are several studies showed that the autophagy is required for activation of PSC (Endo et al., 2017). Saikosaponin A inhibits the activation of PSCs by suppressing autophagy and the NLRP3 (nucleotide-binding domain leucine-rich repeat and pyrin domain containing receptor 3) inflammasome (Cui et al., 2020). Additionally, inhibiting autophagy can also suppress pancreatic fibrosis through promoting ECM degradation by decreasing the expression of TGF-β1 and increasing MMPs/TIMPs ratio (Li et al., 2018a). Retinoblastoma coiled coil protein 1-induced autophagy can facilitate PSC activation and pancreatic fibrosis in CP (Li et al., 2018c; Zhang et al., 2021). Contrarily, PDGF inhibits autophagy in HSC and increases the release of extracellular vesicle (EV) (Gao et al., 2020), while the release of EV can promote the interaction between cells and fibrosis (Xu et al., 2018), suggesting that autophagy in HSC alleviated liver fibrosis by reducing the release of HSC-derived EV (Gao et al., 2020). The role of autophagy is different in various cell types linked to liver diseases. Targeting autophagy has been considered as a potential strategy to treat acute liver injury and non-alcoholic fatty liver disease (Allaire et al., 2019). However, the role of autophagy on PSC activation and pancreatic fibrosis and the therapeutic value of VD-induced autophagy need to be further clarified.

## 6 Conclusion and Perspectives

VD deficiency is prevalent in patients with CP which is associated with the risk and the prognosis of CP. VD supplementation is expected to reduce the risk and improve the prognosis of CP. VD plays a variety of biological functions in the body and has been widely used in the study of inflammatory diseases. VD and its analogues have been confirmed to inhibit PSC activation and reduce ECM deposition, thereby alleviating pancreatic fibrosis. These evidences suggest that VD may be a potential anti-fibrotic therapeutic agent for CP. However, some meta-analyses and clinical studies have found that the relationship between VD deficiency and CP is unclear. At present, large-scale and high-quality prospective studies are needed to confirm the exact role of VD on anti-fibrosis in CP. In the future, more clinical trials of VD and its analogues for the treatment of CP should be carried out, especially RCT studies. There is still much effort to be done to translate clinical trials into clinical practice. These efforts will contribute to the development of an economical and effective agent for the treatment of CP.
